# Photoactive nanoarchitectures based on clays incorporating TiO_2_ and ZnO nanoparticles

**DOI:** 10.3762/bjnano.10.114

**Published:** 2019-05-31

**Authors:** Eduardo Ruiz-Hitzky, Pilar Aranda, Marwa Akkari, Nithima Khaorapapong, Makoto Ogawa

**Affiliations:** 1 Materials Science Institute of Madrid, CSIC, C/ Sor Juana Inés de la Cruz 3, Cantoblanco, 28027 Madrid, Spain; 2 Laboratory of Nanomaterials and Renewable Energy Systems. Research and Technology Center of Energy, Borj-Cedria Science and Technology Park, BP 95, 2050 Hammam-Lif, Tunisia; 3 Materials Chemistry Research Center, Department of Chemistry and Center of Excellence for Innovation in Chemistry, Faculty of Science, Khon Kaen University, Khon Kaen 40002, Thailand; 4 School of Energy Science and Engineering, Vidyasirimedhi Institute of Science and Technology (VISTEC), 555 Moo 1 Payupnai, Wangchan, Rayong 21210, Thailand

**Keywords:** clays, nanoarchitectures, photocatalysts, titanium dioxide, zinc dioxide

## Abstract

Thought as raw materials clay minerals are often disregarded in the development of advanced materials. However, clays of natural and synthetic origin constitute excellent platforms for developing nanostructured functional materials for numerous applications. They can be easily assembled to diverse types of nanoparticles provided with magnetic, electronic, photoactive or bioactive properties, allowing to overcome drawbacks of other types of substrates in the design of functional nanoarchitectures. Within this scope, clays can be of special relevance in the production of photoactive materials as they offer an advantageous way for the stabilization and immobilization of diverse metal-oxide nanoparticles. The controlled assembly under mild conditions of titanium dioxide and zinc oxide nanoparticles with clay minerals to give diverse clay–semiconductor nanoarchitectures are summarized and critically discussed in this review article. The possibility to use clay minerals as starting components showing different morphologies, such as layered, fibrous, or tubular morphologies, to immobilize these types of nanoparticles mainly plays a role in i) the control of their size and size distribution on the solid surface, ii) the mitigation or suppression of the nanoparticle aggregation, and iii) the hierarchical design for selectivity enhancements in the catalytic transformation and for improved overall reaction efficiency. This article tries also to present new steps towards more sophisticated but efficient and highly selective functional nanoarchitectures incorporating photosensitizer elements for tuning the semiconductor–clay photoactivity.

## Review

### Introduction: immobilization of nanoscale TiO_2_ and ZnO on clay minerals

**Figure 1:**
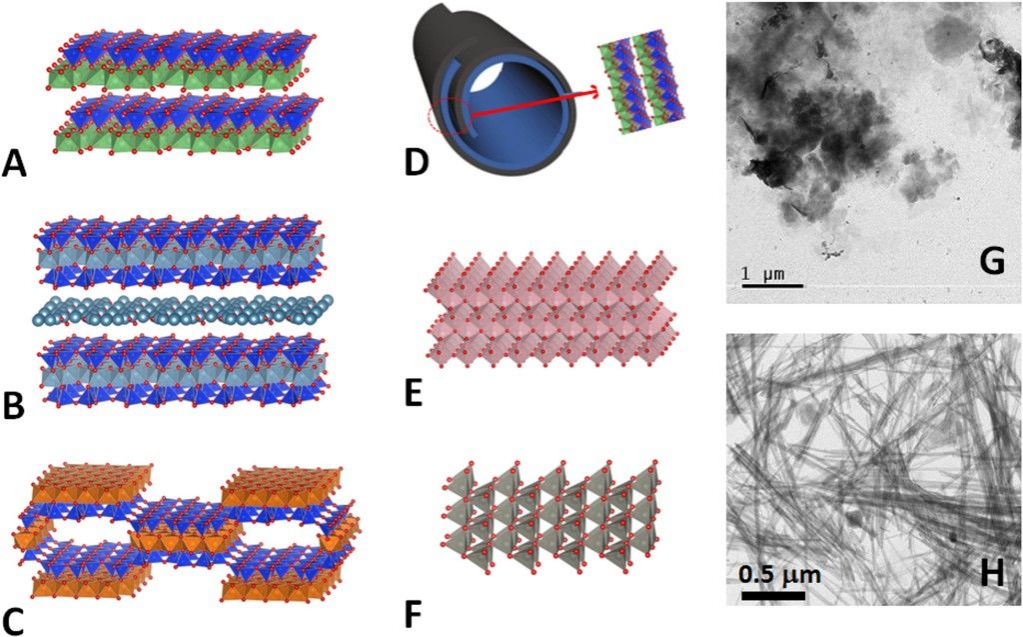
Schematic representation of the crystal structures of the following clay minerals: kaolinite (A), montmorillonite (B), sepiolite (C), halloysite nanotubes (HNT) (D), and the metal oxides, anatase (E) and wurtzite (F), obtained by applying the VESTA software using the following color codes: silicon oxide tetrahedron – blue: Si, red: O. In kaolinite and halloysite – aluminium oxide-hydroxide octahedron: green, red: O; magnesium oxide octahedron: brown, red: O. In montmorillonite – Al,Mg octahedron: pale blue; Ti: pink; Zn: gray. Panel D shows the nanotubular morphology of HNT resulting from the rolling of layers of 1:1 aluminium phyllosilicate with the same structural arrangement as kaolinite. TEM images of (G) montmorillonite and (H) sepiolite clay minerals.

Nanoarchitectonics is a term coined by Japan's National Institute for Materials Science (NIMS), which refers to the nanoscale design of complex materials through a deep and detailed understanding of the interactions between individual nanostructures and their organization [1–3]. 2D nanoarchitectures have been recently reviewed by Ariga and collaborators [4–6]. Among 2D solids, clay minerals have been widely studied as versatile components for the preparation of functional nanoarchitectures by means of their assembly with diverse active compounds including nanoparticles (NPs) of variable nature, such as metal-oxide NPs, which is of great interest for many diverse applications [7–11]. As it is well known, clay minerals are a big family of silicates showing diverse structural arrangements and morphologies (Figure 1) with topologies able to accommodate a variety of NPs of semiconductors such as TiO_2_ and ZnO. TiO_2_ and, to a minor extent, ZnO NPs in the form of anatase and wurtzite phases (Figure 1E and 1F, respectively), are semiconducting materials that have been assembled at the nanometer scale with clay silicates and deeply investigated due to their useful properties for various applications, including heterogeneous photocatalysis, antibacterial activity, and water splitting [12–20]. Both semiconducting solids are more efficient as photocatalysts than the corresponding bulk TiO_2_ and ZnO powders when they are present as NPs. This fact could be simply explained by the fact that the smaller particles normally have a larger surface-to-volume ratio.

Many studies have focused on the photocatalytic activities of TiO_2_ and ZnO supported on clays, clay minerals and related solids that include layered double hydroxides, such as hydrotalcite, and layered polysilicates, such as magadiite. However, this article will focus only on materials based on clay minerals. The emphasis of these studies has been the photodegradation of diverse organic compounds, including the photodecolorization of dyes such as methylene blue (MB), methyl green (MG), acid red G, acid yellow 11, acid orange 11, and Congo red, in water, as well as other photo-applications, for instance, water splitting under UV or visible/solar light irradiation (Table 1). Among the 2D clay-based solids (layered silicates), montmorillonite and other smectites used for assembly with diverse NPs exhibit excellent adsorption, rheological, ion-exchange, and swelling properties as well as a large relative surface area for incorporating the NPs. Kaolinite clay presents lower values of ion-exchange capacity and a smaller relative surface area as, in general, the interactions only involve its external surface. However, the latter aluminosilicate shows a chemical inertness that is useful for its use as support of the semiconducting NPs considered here.

**Table 1:** Selected examples of catalytic applications of TiO_2_@clay and ZnO@clay nanoarchitectures.

clay-based nanoarchitectures		photodegradation	other applications
clay component	semiconductor component		

kaolinite	TiO_2_	Kutláková et al. (2011) [94]; Zhang et al. (2011) [95]; Chong et al. (2009) [96]; Barbosa et al. (2015) [98]	CO_2_ reduction, Kočí et al. (2011) [97]
kaolinite	ZnO	M. Kutláková et al. (2015) [172]	antibacterial activity, Dĕdková et al. (2016) [173]
halloysite	TiO_2_	Papoulis et al. (2013) [174]; Wang et al. (2011) [123]; Li et al. (2015) [175]; Du et al. (2014) [176]; Papoulis et al. (2010) [114]	—
hectorite	TiO_2_	Ma et al. (2009 & 2010) [103,108], Kibanova et al. (2009) [101]; Belessi et al. (2007) [102]	—
Laponite^®^	TiO_2_	Zhu et al. (2002) [177]	—
Ce–Ti-pillared Laponite^®^	TiO_2_	Lin et al. (2010) [178]	—
Zr–Ti-pillared Laponite^®^	TiO_2_	Lin et al. (2011) [179]	—
stevensite	TiO_2_	Bouna et al. (2014) [180]	—
beidellite	TiO_2_	Rhouta et al. (2015) [99]	—
Ti-pillared beidellite	TiO_2_	—	cracking of cumene, Swarnakar et al. (1996) [181]
TiO_2_-pillared saponite	TiO_2_	—	degradation of NO_x_ gas, Nikolopoulou et al. (2009) [182]
montmorillonite, bentonite and related smectites	TiO_2_	Sun et al. (2015) [105]; Manova et al. (2010) [106]; Rossetto et al. (2010) [104]	—
montmorillonite, bentonite and related smectites	ZnO	Fatimah et al. (2011) [89]; Khumchoo et al. (2016) [46]; Ye et al. (2015) [120]; Akkari et al. (2016) [118]; Xu et al. (2014) [124]	
montmorillonite and related smectites	TiO_2_/ZnO	Bel Hadjltaief et al. (2016) [158]; Tobajas et al. (2017) [159]	
montmorillonite (CTA-organoclay)	ZnO	—	antibacterial activity, Gu et al. (2015) [117];
lightweight expanded clay aggregates (LECA)	TiO_2_/ZnO	—	removal of ammonia from wastewater; Mohammadi et al. (2016) [161]
montmorillonite–kaolinite	TiO_2_	—	removal of Pb(II), Cu(II), Zn(II), and Cd(II); Đukić et al. (2015) [183]
bentonite	TiO_2_	—	Degradation of volatile organic compounds (VOCs); Mishra et al.*(*2018) [184]
rectorite	TiO_2_	Bu et al. (2010) [185]; Zhang et al. (2011) [186]; Yang et al. (2012) [187]	—
rectorite	ZnO	Li et al. (2014) [188]	—
rectorite	TiO_2_/ZnO	Wang et al. (2018) [163]	—
sepiolite	TiO_2_	Aranda et al. (2008) [109];Suárez et al. (2008) [112]; Ökte & Sayınsöz (2008) [113]; Du et al. (2015) [189]^a^; Zhou et al. (2017) [190]	photoreforming of methanol, Pérez-Carvajal et al. (2016) [131]^b^
sepiolite	ZnO	Xu et al. (2010) [90]; Akkari et al. (2016) [118]	removal of H_2_S by chemisorption, Portela et al. (2015) [122]
sepiolite	TiO_2_/ZnO	Vaizoğullar (2017) [160]	
palygorskite	TiO_2_	Zhang et al. (2011) [191]^c^; Bouna et al. (2011) [111]; Papoulis et al. (201) [115] Stathatos et al. (2012) [116]	degradation of NO*_x_* gas, Papoulis et al. (2010) [114]
palygorskite	ZnO	—	antibacterial activity, Huo and Yang (2010) [121]

^a^containing Ag_2_O/TiO_2_; ^b^Pt- or Pd-doped TiO_2_; ^c^containing SnO_2_/TiO_2_.

According to the “Web of Science” (WoS) [21] around 10,000 papers have been published in the last decade in connection with the topic of TiO_2_ NPs used as photocatalysts, indicating the high interest in the use of these materials for this type of applications. In fact, titanium dioxide (anatase phase) can be considered the most extensively studied solid among the diverse transition-metal oxides and transition-metal chalcogenides investigated with that focus over the last decades. However, TiO_2_ has disadvantages such as limited activity together with a reduced sensitivity to sunlight. Hence, alternative semiconductors such as ZnO are increasingly investigated for processes concerning environmental remediation, antibacterial activity and chemical technologies for hydrogen production and synthesis of organic compounds [22]. Anyway, according to WoS, in the given period TiO_2_ NPs appear to be cited ten times more often than ZnO NPs regarding their use as photocatalysts.

One of the main applications of clay–semiconductor materials is the mineralization of organic pollutants, which represents an ideal solution for the remediation of wastewater contaminated with diverse organic species. This process consists in the oxidative decomposition of organic pollutants to non-toxic inorganic species such as carbon dioxide, avoiding the formation of any kind of residual sludge [23]. In fact, advanced oxidation processes (AOP) might be considered as promising technologies for the sustainable removal of pollutants from urban, industrial and agricultural wastewater. They are based on the in situ generation of reactive species as hydroxyl radicals (OH^•^) with high oxidizing capability [24]. AOP include Fenton and photo-Fenton reactions based on the combination of chemical oxidants, e.g., hydrogen peroxide, and UV irradiation, and other photocatalytic reactions assisted by semiconductor photocatalysts. The use of TiO_2_ and ZnO NPs, particularly the anatase and wurtzite phases, as heterogeneous photocatalysts attracted great attention over the last years. Atmospheric oxygen is used as oxidant to achieve complete mineralization of harmful organic compounds, such as pesticides, phenols, dyes, and pharmaceuticals drugs to water, CO_2_, and non-toxic inorganic residues under solar light or UV irradiation. Advantages such as low cost and chemical stability are essential requirements of this type of photocatalysts [25–26]. In addition, highly toxic heavy metals such as Cr(VI) and Hg(II) can be removed from aqueous environments by photoreduction employing NPs as heterogeneous photocatalysts leading to insolubilization [23,27–29].

Based on the redox and photoactive properties, TiO_2_ and ZnO are the most commonly reported nanoparticulated materials for photocatalytic applications as they can be considered to be chemically stable and easily available (inexpensive commercial products) [12–13,30–33]. In this context, ZnO appears as a better candidate for water cleaning compared to the more commonly used TiO_2_. In fact, ZnO shows a wider absorption in the visible-wavelength region than TiO_2_, which is advantageous [34–43].

A possible higher catalytic activity of ZnO compared to TiO_2_ has also been discussed. However, contradictory arguments and results have been reported [20]. The origin of the experimentally observed differences in the photoactivity are not yet clear due to the effect of complex parameters including light absorption, charge recombination, changes in the available surface area and chemical reactivity [44–45]. Another characteristic of ZnO is its lower chemical stability compared to TiO_2_, particularly in acidic solution, which can be a drawback for practical applications [46–47].

By changing morphology, doping, and conformation, i.e. as films, of the nanoparticles the intrinsic opto-electronic properties of the semiconductor as well as its chemical stability and surface reactivity can be modified [48–49]. TiO_2_ and ZnO NPs with different shapes, sizes and exposed crystal facets were assembled to yield hollow particles, fibers, nanosheets, nanowires, nanorods, nanoflowers and nanobelts through various synthesis routes including template synthesis [50–53]. Controlling the NP morphology can be advantageous for the following purposes: i) to increase the available surface area for the reactant access, ii) to obtain stable aqueous suspensions for efficient light transmission (or to suppress light scattering), and iii) to expose specific catalytically active facets. Synthesis strategies of hierarchically designed TiO_2_ and ZnO nanoarchitectures with controlled morphology, crystallinity, anisotropic shape, and composition have been investigated recently. The aim was to obtain accessible and interconnected porous networks with increased specific surface area for the adsorption of reactants and diffusion of the products [54].

Photocatalysts need to be removed and recovered from the suspension after use. When the photocatalysts are present as nanoscale particles, their aqueous suspensions become more stable, reducing unfavorable effects such as scattering. However, the recovery of the NPs, e.g., through filtration, can be very difficult, which may add substantial costs to industrial processes involving these materials. In addition, the tendency of the particles to aggregate, especially at high concentrations, may cause changes in the transparency and viscosity of the suspensions.

To overcome these drawbacks, the development of active photocatalysts based on supported NPs appears as a promising solution to these problems. Hence, the development of efficient nanoarchitectured clay–semiconductor NP materials is an attractive option.

TiO_2_ and ZnO NPs assembled with different inorganic solids (substrate or matrix components) are advantageous not only for photocatalytic purposes but also for applications as pigments and cosmetics, where colloidal and surface characteristics play a significant role. TiO_2_ and ZnO NPs have been prepared in the presence of inorganic matrices by in situ formation in the available nanoscale spaces of the solid substrates, resulting in controlled size and shape of the supported TiO_2_ and ZnO NPs. The involved inorganic matrices can be of very diverse nature such as silica and silicates (mesoporous silicas, zeolites, clays, and clay minerals), carbonaceous materials (carbon nanotubes, graphene, graphene oxide, and activated carbon), layered double hydroxides, layered polysilicates (magadiite and kenyaite), and metal organic frameworks. The role of the inorganic matrices in the assembly of the semiconductor NPs [55–59] is: i) to control the particle size and the size distribution of the growing NPs; ii) to immobilize the NPs either on the external surface or within nanoscale spaces, e.g., pores and intracrystalline cavities; iii) to diminish/to avoid NPs aggregation; iv) to suppress the NPs dissolution; v) to yield stable suspensions more suitable for photocatalytic reactions; vi) to facilitate percolation in membrane or column designs for the easier separation and collection of products; and vii) to enable molecular recognition in photocatalysis through the well-defined nanopores in the inorganic component.

Well-defined nanoporous solids such as zeolites and mesoporous silicas have been used as templates in the growth of NPs with precisely controlled particle size and shape replicating size and shape of the pores. Thus, a novel class of nonlinear optical materials based on host–guest composites has been prepared using zeolites as inorganic crystalline hosts [60–66]. Nanoarchitectures composed of zeolites and mesoporous silica and TiO_2_ or ZnO NPs have been reported as efficient photocatalysts as well as photoluminescent materials [67–69]. The formation of TiO_2_ and ZnO NPs on the external surface, in addition to the NPs confined in the nanopores, is still an important challenge. Recently, the size-controlled synthesis of TiO_2_ NPs within mesoporous silica (SBA-15) has been reported, where, according to Vibulyaseak and co-workers [70], the NPs formation occurred exclusively in the mesopores.

The aim of this work is to summarize and critically discuss the different experimental options in the use of TiO_2_ and ZnO NPs, assembled with clay minerals and related solids, emphasizing on their structural and textural characteristics in relation to their photocatalytic activity.

### Synthetic strategies for the preparation of TiO_2_ and ZnO nanoarchitectures: modulation of their physical and chemical characteristics

As introduced above, different functional nanoarchitectures for various applications have been synthesized from clay minerals taking advantage of their natural abundance and eco-friendly nature, as well as their unique structural and textural features. It is well known that these silicates show different nanostructures and particle shapes, such as lamellar (smectites and kaolinites), fibrous (sepiolite and palygorskite), and tubular (halloysite) morphologies (Figure 1). Mainly on the basis of the ion-exchange of their interlayer inorganic cations and to the presence of reactive hydroxyl groups at their external surfaces, it is possible to modify in a controlled manner the surface characteristics of clay minerals introducing new suitable functions leading to hierarchically structured nanoarchitectures [8,10–11].

A useful strategy to enhance the photocatalytic activity of metal-oxide NPs considered here consists in their distribution as homogenously as possible on the surface of clay minerals acting as supports and provided with large specific area and porosity. Among the clay materials (Figure 1), layered silicates such as smectites are of particular interest as they have been largely used in adsorption and catalysis applications due to their valuable properties as expandable interlayer space, low cost and environmentally friendly nature [71]. Similarly, fibrous silicates, i.e., palygorskite and sepiolite, are characterized by a large specific surface area and microporosity, as well as the presence of external silanol groups, which can immobilize species including NPs of diverse nature [11,72]. Halloysite (Figure 1D) is a layered aluminosilicate with a silica/alumina composition similar to that of kaolinite that can be present as a tubular clay (halloysite nanotubes, HNTs) with diameters of 50–80 nm (external) and 10–15 nm (internal), and a typical length of ca. 1000 nm [73–75]. The external surface of HNTs is composed of siloxane groups (Si–O–Si) while the internal surface is covered by aluminol groups (Al–OH) with a structural arrangement similar to that of the kaolinite 1:1 phyllosilicate (Figure 1D). These groups able to interact with diverse compounds entering the cavities, facilitating their immobilization [76–79]. The morphology of HNTs yields some advantages with regard to the development of new architectures, including the immobilization of TiO_2_ and ZnO NPs.

Semiconductor NP–clay nanoarchitectures prepared from natural or synthetic clay minerals have been extensively investigated, involving both the assembly of already formed metal-oxide particles as well as the in situ formation of NPs [80–83]. The assembly of TiO_2_ and ZnO NPs with clays of different characteristics takes place mainly on the external surfaces. However, in materials produced from layer silicates of the smectite group, the interlayer space may also be involved. In this case, it is important to control the synthesis procedures in order to obtain the desired surface properties of the NPs, as well as suitable size and shape and nanopore characteristics in the resulting pillared layered structures.

The formation of house-of-cards-like structures during the preparation of ZnO NPs in the presence of smectites as a result of the re-stacking of the exfoliated nanosheets has been reported [84]. Due to the variety of hierarchical structures and particle locations (at the external surface or in the interlayer space), the resulting particle size distribution of ZnO NPs can be very wide, affecting the physio-chemical characteristics of the resulting clay-based nanoarchitectures [85–87].

Nanotubular halloysite and microporous fibrous silicates such as sepiolite and palygorskite can be also considered as good candidates for the size-controlled growth of NPs, due to their well-defined nanopore structure and other surface characteristics. However, in the case of sepiolite and palygorskite, the sizes of the available nanospaces are too small for the generation of TiO_2_ and ZnO NPs within the nanopores [72]. In this case, mesopores, which can be ascribed to inter-fibre regions, could facilitate the growth of the semiconductor NPs. This happens in the same way as that occurring at the external surface of the fibrous clay minerals with the participation of surface Si–OH groups for anchoring the TiO_2_ and ZnO NPs.

The assembly of TiO_2_ and other types of semiconducting NPs takes place very often on the external surface of clay minerals [8,11]. The particle shape and size have been evaluated by transmission electron microscopy (TEM) and X-ray diffraction (XRD) using the Scherrer equation, in addition to the spectroscopic information obtained from the shift of the UV–vis absorption band to a shorter wavelength region, showing quantum-size effects. The advantages of clay minerals acting as supports for TiO_2_ and ZnO NPs are the presence of surface electrical charge and/or the elevated concentration of hydroxy groups on the available surface, which can have an important influence on the NPs immobilization as well as on the structural stability during the photocatalytic reactions. In addition, the immobilization of NPs on clay surfaces is a key advantage for the easier recovery of the photocatalyst from the reaction medium compared to bare NPs [88–93].

Clay-based nanostructured materials prepared by in situ formation of NPs can be achieved by applying various procedures such as impregnation by precipitation, sol–gel, solvothermal and microwave-assisted reactions. As already indicated, clay-based nanoarchitectures containing TiO_2_ NPs (anatase phase) are currently the most extensively studied clay–semiconductor systems for photocatalysis applications. Various procedures have been reported to produce kaolinite clay mineral fully coated with TiO_2_ NPs [94–98]. An example of these methods is the in situ formation of titanium dioxide and its anchorage on the external kaolinite surface through sol–gel methods as, for instance, one based on the controlled hydrolysis titanium(IV)-*n*-butoxide in ethanol, resulting in heterocoagulation with kaolinite in aqueous suspensions [96]. However, kaolinite can be expanded by the intercalation of polar molecules such as urea, dimethyl formamide and dimethyl sulfoxide (DMSO), which could facilitate the access of other compounds to the interlayer region of this phyllosilicate. Németh and co-workers [85] claimed the generation of ZnO NPs in the interlamellar space of kaolinite following an alkaline hydrolysis of the clay treated with a solution of Zn-cyclohexanebutyrate dihydrate in DMSO. From the XRD patterns, it has been found that at low ZnO loading confined NPs of a very small size (1–2 nm) are produced, whereas at high ZnO loading, a part of the ZnO NPs grew at the external surface of kaolinite. The absorption onset of ZnO in layered clay minerals showed a strong blue-shift compared to bare ZnO prepared at the same precursor concentrations. In kaolinite, the particle size of ZnO was larger and the intercalation ratio was smaller than in montmorillonite 2:1 (a typical smectite clay mineral). In this last case, the average particle size of ZnO (2.6–13.0 nm) obtained from the same concentration of the precursor was affected by the cation-exchange capacity of the clay minerals [85].

Layered clay minerals belonging to the smectite group, such as montmorillonite, hectorite, stevensite and beidellite [99–108], as well as fibrous silicates such as sepiolite and palygorskite [109–116] have been also assembled with TiO_2_ NPs yielding various clay-based nanoarchitectures with photocatalytic activity (Table 1). For instance, a method to develop TiO_2_@hectorite as multilayer ﬁlms using layer-by-layer self-assembly has been reported by Ma and co-workers [103]. TiO_2_ NPs have been also precipitated in the presence of layered silicates such as montmorillonite and hectorite using Ti alkoxides or TiCl_4_ and TiCl_3_ as precursors. Similarly, ZnO NPs have been assembled with diverse smectites [46,89,117–120] and fibrous clay minerals [87,90,118,121–122].

**Figure 2:**
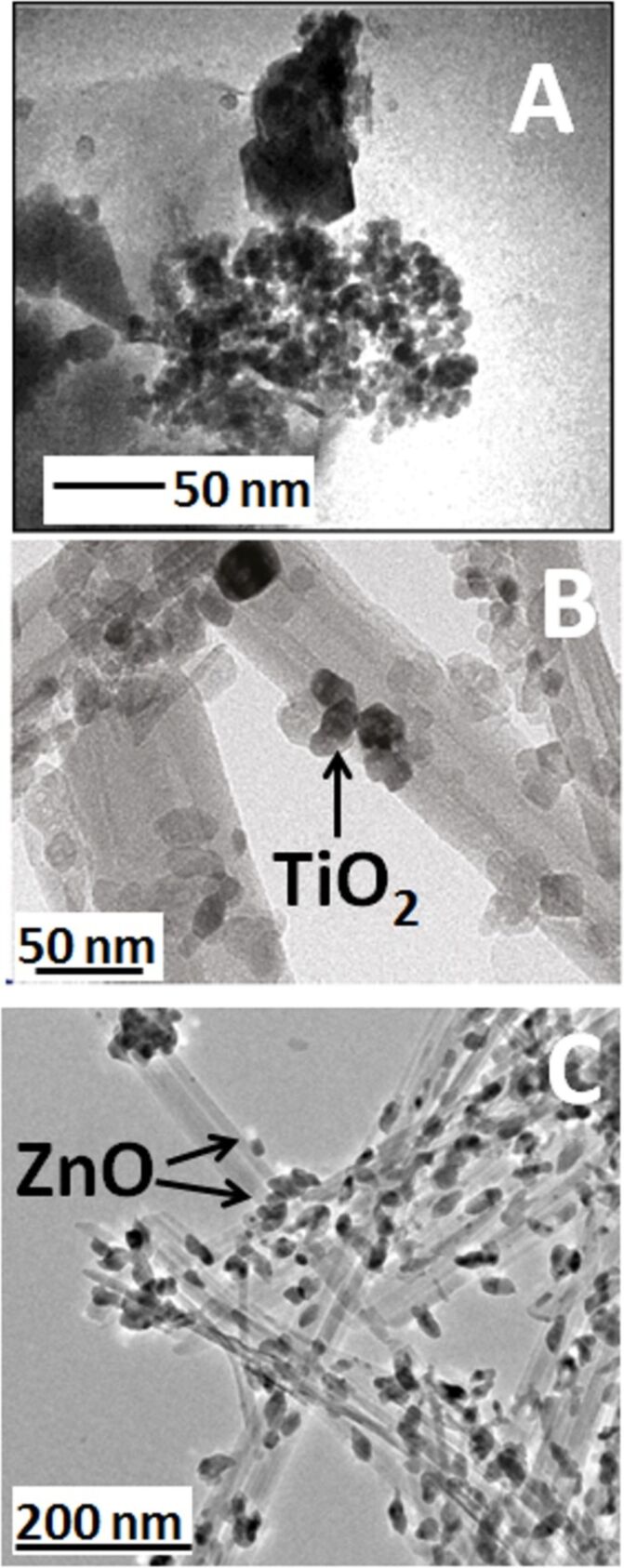
TEM images of (A) ZnO NPs on montmorillonite with nanocrystal aggregation; reprinted with permission from [85], copyright 2004 American Chemical Society; (B) TiO_2_@HNTs nanoarchitecture showing the titania NP assembled inside the lumen of the halloysite tubes; adapted with permission from [123], copyright 2011 American Chemical Society; and (C) ZnO@sepiolite where the ZnO NPs were generated on the external surface of the fibrous clay from zinc acetylacetonate following the protocol described in [87].

The characteristics of the NPs on clay minerals vary depending on the preparation conditions. For instance, in the case of TiO_2_@clays formed by the hydrolysis at 90 °C of titanium(IV) oxysulfate in a kaolinite suspension, anatase NPs of 18 nm size were formed. In this case, a preferential growth at the edges of platy aluminosilicate particles was observed [94,97]. This preference can be tentatively ascribed to the anchorage of TiO_2_ NPs through the aluminol groups located at the kaolinite edges. These NPs grew from 6 to 18 nm during calcination at 500–700 °C with a phase transition from anatase to rutile taking place at ca. 650 °C [96]. Layered silicates, such as kaolinite and montmorillonite, also stabilize the formation of ZnO NPs (Figure 2A) from zinc cyclohexanebutyrate hydrolyzed in dimethyl sulfoxide. The particle size is clearly influenced by the nature of the clay [85]. In the case of HNTs, TiO_2_ NPs of 5–15 nm size were formed inside the halloysite tubes (lumen) as observed by TEM (Figure 2B) [123]. Moreover, Papoulis and co-workers [114] have been reported that the particle size of TiO_2_ NPs is about 3–15 nm in the macropores of the clay, which corresponds to the central hole in HNTs, leading to the blockage of the lumen. The particle size of titania NPs homogeneously deposited on the external surface of palygorskite fibrous clay was 10–30 nm as detected by SEM [114]. In another example, ZnO NPs from zinc acetate ca. 20 nm were exclusively located at the external surface of the palygorskite silicate fibres [121]. ZnO can be also directly formed on the external surface of sepiolite fibres (Figure 2C), using zinc acetylacetonate as precursor [87].

As reported by Fatimah et al. [89], ZnO@montmorillonite materials can be synthesized from a Zn solution and cetyltrimethylammonium (CTA)-montmorillonite organoclays. In these materials, the bandgap energy of ZnO is decreased compared to bare ZnO NPs, which results in a faster photodegradation of MB. In experiments to prepare ZnO@clay nanoarchitectures using smectites such as natural montmorillonite, synthetic saponite, as well as the corresponding CTA-smectites, Khaorapapong and co-workers [46,92–93] synthesized diverse photocatalysts where the ZnO NPs were formed on the inner and/or outer surfaces of the CTA-smectites. The photoluminescence at visible wavelengths (blue and green emission at around 436–438 nm and 544–548 nm) of ZnO hybridized with CTA-smectites varies depending on the ZnO loading. This was attributed to defects, such as oxygen vacancies in ZnO, and trapped surface charges. The photoluminescence intensity of ZnO in saponite and CTA-saponite was stronger than in montmorillonite and CTA-montmorillonite, suggesting that the iron atoms in montmorillonite play a significant role through the quenching of excited states. In the photodegradation experiments, the ZnO–smectite nanoarchitectures show a longer life time at low pH values than bare ZnO. This was attributed to the dissolution of ZnO in acidic solution, which is suppressed by the hybridization with smectites [46,92–93]. CTA-smectites treated with a hydrothermal solution intercalation method at 70 °C for 10 h lead to ZnO@CTA-montmorillonite where the ZnO NPs are either embedded in the interlayer space of the organoclays or dispersed on its external surface. These nanomaterials can be applied as antibactericide. It has been observed that they destroy the cellular surface structure of *Microcystis aeruginosa*, and also inhibit the physiological activity of *M. aeruginosa*, when exposed to UV light [117]. Other alternative approaches include the direct precipitation of the ZnO from Zn salts in presence of bentonite dispersed in ethanol [124] or the association of ZnO NPs with Laponite^®^ using poly(vinyl alcohol) as binder agent [125]. In both cases, the resulting materials show photocatalytic activity and are easily recoverable from the reaction medium.

**Figure 3:**
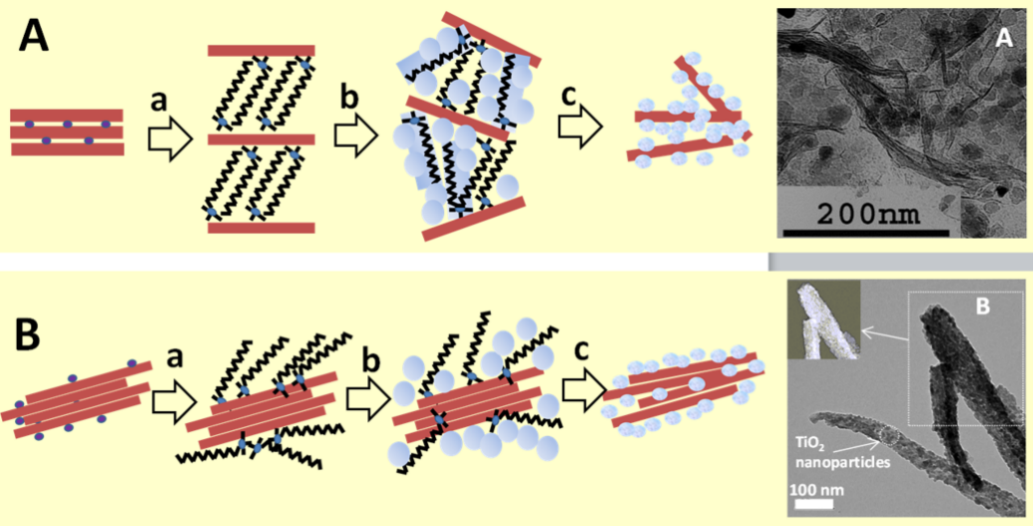
Synthesis of clay–semiconductor nanoarchitectures by the “organoclay colloidal route” involving either smectites (A) or fibrous clays (B) in the following steps: a) replacement of inorganic cations by alkylammonium ions forming the intermediate organoclay, which is treated with metal-oxide precursors being transformed (b) into intermediate compounds that after calcination (c) finally yield the nanoarchitecture containing the photoactive semiconductor. TEM Images (on the right) of A: ZnO@smectite from Gafsa, where ZnO NPs were previously prepared from Zn acetate [118], and B: TEM of TiO_2_@sepiolite, where TiO_2_ NPs were prepared from titanium isopropoxide; reprinted with permission from [109], copyright 2008 American Chemical Society.

The so-called “organoclay colloidal route” [71,109,126] represents an innovative approach to prepare, under mild conditions, porous nanoarchitectures from alkylammonium-exchanged smectite clays combined to metal-oxide NPs already synthesized or formed in situ by incorporation of the corresponding precursors (alkoxides, salts in alkaline medium and metal complexes) as schematized in Figure 3. Of particular relevance is the irreversible delamination of the 2:1 charged layered silicates, e.g., smectite clay minerals, taking place during the heterocoagulation of hydrolyzed alkoxides previously incorporated in the surfactant–clay interface, as it was first reported by Letaïef and Ruiz-Hitzky [126–127]. In the same way, organoclays dispersed in an organic solvent can facilitate the incorporation of already formed metal-oxide nanoparticles, leading to a loss of the stacking order in the silicate layers due to the assembly with ZnO NPs [118].

The protocol schematized in Figure 3A, a very convenient pathway to produce functional nanoarchitectures by using alkylammonium-exchanged layered clays, has been applied to produce TiO_2_@clay and ZnO@clay materials. Following this approach, micro- and mesoporous clay–semiconductor nanoarchitectures with high pore volume and increased specific surface area due to clay delamination have been obtained. In some of these materials, the TiO_2_ and ZnO NPs generated from the corresponding alkoxides, salts or coordination complexes, remain associated with the delaminated clay sheets, resulting in stable and efficient photoactive catalysts of particular interest for the removal of organic pollutants from wastewater [84,87,89,106–107,118,128]. Akkari and co-workers [118] have recently applied this procedure to assemble ZnO NPs, previously synthesized by hydrolysis of Zn acetate, with organoclays derived from two different smectites (Figure 3A), leading to ZnO@smectite nanoarchitectures in which the delaminated silicate remains associated with ZnO NPs of 7–10 nm size. The specific surface area values are of the order of 50–100 m^2^/g whereas the ZnO NPs alone exhibit values below 15 m^2^/g. The mesoporosity (ca. 0.25 cm^3^/g total porosity) together the photoactivity of the ZnO NPs make these materials suitable photocatalysts for the removal of organic dyes from water [118]. ZnO–clay nanoarchitectures have been prepared by in situ generation of ZnO NPs using Zn acetylacetonate precursor in isopropanol under reﬂux in the presence of the organoclays, leading to intermediate ZnO@clay organo-heterostructures. After calcination, the organic matter (alkyl groups from the organoclay) is eliminated and the ZnO NPs remain assembled to the clay surface [83]. These materials exhibit good photoactivity useful for the removal of organic pollutants such as pharmaceutical drugs from water.

In recent years, fibrous clays are attracting increasing interest as supports for the assembly with a large variety of nanoparticles in the search of new functional and multifunctional nanostructured materials [11]. The procedures used to assemble TiO_2_ and ZnO NPs with sepiolite and palygorskite include the in situ generation from salts [90,122,129] or alkoxide precursors in presence of organoclays [109,111], directly on the clay surface from precursors dispersed with surfactants [116] or without [129–130], as well as the direct attachment of already formed particles to the clay [118,122]. The characteristics of the resulting materials are strongly influenced by the preparation conditions as the generated NPs show significant differences in size, degree of self-agglomeration as well as dispersion on the surface of the clay, which may influence the resulting properties of the materials.

**Figure 4:**
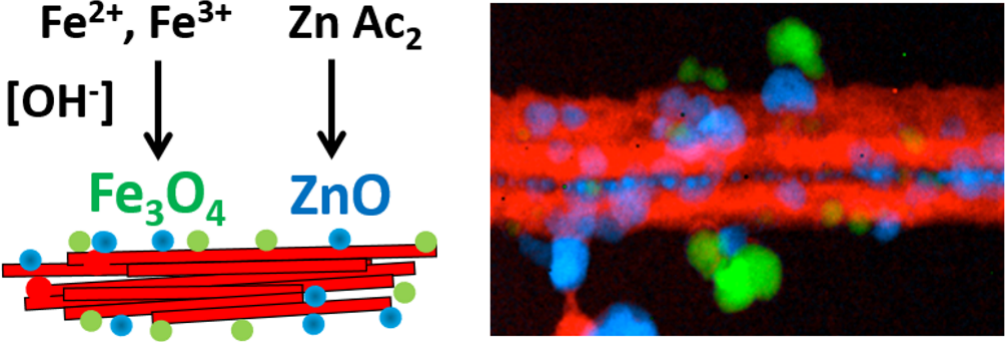
ZnO-Fe_3_O_4_@sepiolite nanoarchitecture prepared in two steps: First, the fiber clay is modified by assembly of magnetite NPs. After that, the ZnO NPs are added yielding a magnetic photocatalyst. The STEM images on the right shows the silicate component (red), the magnetite NPs (green) and the ZnO NPs (blue) analyzed with an EDAX detector and a Gatan Tridiem energy filter; reprinted with permission from [133], copyright 2017 Elsevier.

The principle of using the interfaces in layered clays is also applicable to fibrous clays with the generation of NPs homogeneously distributed on the surface of sepiolite or palygorskite [109,111,118]. Figure 3 also shows TEM images of TiO_2_ produced in a controlled sol–gel process on the external surface of sepiolite modified with cetyltrimethylammonium ions. A coverage of the silicate surface by small NPs (4–8 nm diameter) suitable for photocatalytic applications is clearly seen [109]. An advantage of the organoclay colloidal route is the possibility to incorporate NPs of different nature, in one or multiple steps, which can be of interest for the introduction of diverse functionalities in the resulting nanoarchitectures [131–132]. The incorporation of various types of NPs using neat clay and applying a two-step synthesis has been reported. A recent example of this refers to the incorporation of ZnO nanoparticles to a Fe_3_O_4_-sepiolite nanoarchitecture previously prepared by in situ formation of superparamagnetic iron-oxide nanoparticles on the external surface of sepiolite fibres. The resulting ZnO–Fe_3_O_4_@sepiolite nanoarchitecture exhibits photoactivity due to the ZnO NPs, and the presence of magnetite NPs facilitates the recovery by the use of a magnet (Figure 4) [133]. Moreover, the presence of iron oxide could be useful also to profit from possible Fenton processes improving the overall photocatalytic efficiency. This opportunity would be of interest for future developments of multifunctional nanoarchitectured photocatalysts.

### Tuning the photoactivity of TiO_2_–clay and ZnO–clay nanoarchitectures: catalytic applications and perspectives

Nanoparticulated TiO_2_ has almost the same bandgap characteristics than ZnO, with bandgap energies of 3.20 eV and 3.37 eV, respectively [48,134–136]. Therefore, the photocatalytic capability of both types of NPs should be quite similar. Apart from these large bandgap energy values, both metal oxides exhibit a large exciton binding energy, large piezoelectric constants and strong photoluminescence. This is of interest not only for applications as photocatalysts but also as sensors, solar cell devices, disinfectants, and cosmetics [137–138].

As discussed above, the dispersion of the semiconducting NPs on inert porous solids of large specific surface area is considered to be beneficial for the photocatalytic activity. Recently, strategies have been reported to improve the performance of photocatalysts via doping, or the introduction of semiconductor heterojunctions by combining them with transition metals or with other semiconductors. Among them, semiconductor heterojunctions have attracted great attention [139]. The doping of TiO_2_ and ZnO NPs with the aim to conveniently tuning the bandgap energy values can be a suitable option. In this context, it has been verified for both types of NPs, a decrease in the bandgap values by doping with Ag, Pd and other transition metals such as Zr, W, Ce, Sn, Sb and In improve the photoactivity efficiency [140]. Alternatively, combination of TiO_2_ and ZnO with other metal oxides leads to mixed oxide NPs, including the TiO_2_–ZnO compositions, which exhibit alternative interesting semiconductor–semiconductor heterojunctions. Finally, another approach to increase the photo-efficiency of the considered systems is the photosensitization of TiO_2_ and ZnO NPs to obtain visible-light responsive photocatalysts as well as solar-cell components [93,141–142]. These approaches to control the intrinsic characteristics of the NPs with the aim of modulating and improving their photoactivity are discussed below.

**Figure 5:**
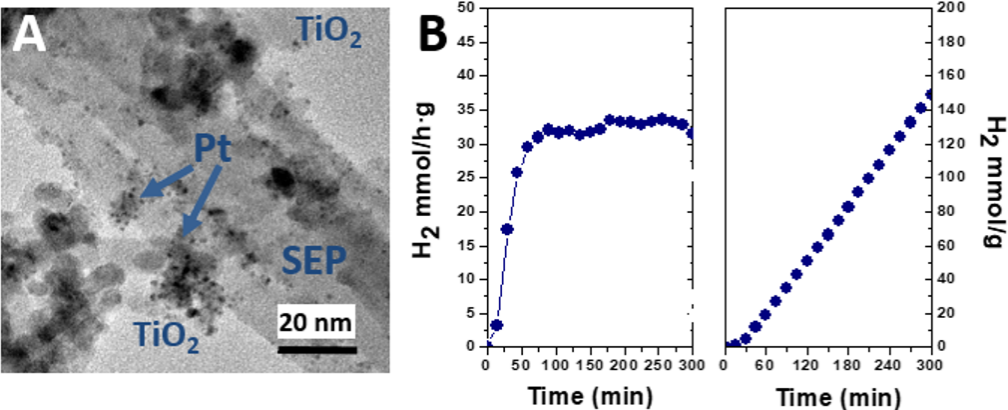
(A) TEM image of the Pt–TiO_2_@sepiolite clay nanoarchitectures prepared by a photodeposition procedure, reprinted with permission from [131], copyright 2015 Elsevier; (B) hydrogen production in methanol photoreforming using this Pt-doped clay nanoarchitecture as catalyst [131].

Li and co-workers [143] have prepared ZnO@kaolinite doped with Pd(II) following a soft chemistry procedure that involves the use of PdCl_2_ and polyvinylpyrrolidone as starting reagents. Interestingly, they reported a considerable increase of the photocatalytic activity in the degradation of methylene blue (MB) in water solution under UV irradiation for Pd–ZnO@kaolinite compared to Pd@ZnO, ZnO@kaolinite, and pure ZnO under equivalent experimental conditions. Pd- and Pt-doped TiO_2_@clay nanoarchitectures based on sepiolite and montmorillonite have been also prepared by applying two different strategies: i) in situ incorporation of the noble-metal precursor (typically acetylacetonate) in the suspension of commercial organoclays (e.g., Cloisite^®^30B and sepiolite Pangel B20, prepared by modification of montmorillonite and sepiolite with cationic surfactants, respectively) during sol-gel process, and ii) selective photodeposition of the noble metal in the previously formed TiO_2_@clay nanoarchitecture [131]. The good dispersion of the noble-metal NPs, clearly revealed by TEM (Figure 5A), leads to efficient nanostructured materials for the photocatalytic production of hydrogen tested in methanol photoreforming. Herein, montmorillonite-based nanoarchitectures are less efficient as hydrogen production catalyst than nanoarchitectures derived from sepiolite. Higher rates of hydrogen production are obtained with the Pt-doped TiO_2_@sepiolite nanoarchitectures obtained by photodeposition (Figure 5B).

Photocatalysts based on Ag-doped ZnO@montmorillonite reported by Sohrabnezhad and Seiﬁ [144] are another example for the enhancement of photocatalytic activity through doping. Silver metal NPs (Ag) were prepared from AgNO_3_ and deposited over ZnO@montmorillonite following a green approach for the Ag^+^ reduction. Here again, the doped nanoarchitecture (Ag–ZnO@montmorillonite) exhibited a better efﬁciency than the corresponding ZnO@montmorillonite and Ag@ZnO samples, in this case tested in the MB removal from water solutions.

Belver and co-workers [145–147] have recently reported that doping of TiO_2_@clay nanoarchitectures with elements such as W, Zr and Ce leads to enhanced solar photocatalytic activity for the removal of organic pollutants such as pharmaceutical drugs, organic dyes, and phenols in aqueous solution. The single-step synthesis yields W–TiO_2_, Zr–TiO_2_ and Ce–TiO_2_ immobilized on the surface of delaminated layered clay derived from montmorillonite. The resulting doped TiO_2_@clay materials showed high specific surface area values and a slight reduction of the TiO_2_ bandgap leading to improved efficiency in the degradation of antipyrine, atrazine, rhodamine B and phenol using solar-light irradiation.

Chen and co-workers [148] have also studied the doping of TiO_2_@montmorillonite introducing heteroelements such as C and V. A bandgap reduction to 2.25 eV, corresponding to a light wavelength of 550 nm suitable for a photocatalyst responsive to visible light was reported. The co-doping of N and S [149] and Cu, Ag, and Fe on TiO_2_@bentonite has also been reported [150]. Other authors also reported the modification of the photoactivity characteristics by the deposition of metallic particles such as Ag, V, and Pt on TiO_2_@montmorillonite [151–157].

Novel TiO_2_–ZnO@clay nanoarchitectures have been recently prepared from diverse clay minerals with improved photoactivity in the resulting materials [158–161]. Probably the first contribution was introduced by Bel Hadjltaief and co-workers [158], using a Tunisian Na^+^-smectite treated with titanium(IV) isopropoxide and zinc acetate. This ZnO–TiO_2_@clay material shows that a rapid and complete mineralization of methyl green dye in water can be achieved with the additional of ZnO providing a higher photocatalytic activity to the starting clay or the TiO_2_@clay nanoarchitecture. Related ZnO–TiO_2_@clay materials based on commercially available expanded clay aggregates (LECA), apparently belonging to the smectite family, have been also positively tested as photocatalyst for the removal of ammonia from wastewater (Table 1) [161]. Also, TiO_2_–ZnO@clay nanoarchitectures derived from a smectite (Cloisite^®^30B) have been also prepared by sol–gel reactions involving the delamination of the silicate. The resulting materials show good photocatalytic activity for the photodegradation of pollutants such as the pharmaceutical drugs acetaminophen and antipyrine and the pesticide atrazine [147,159,162]. TiO_2_–ZnO@clay materials have been also prepared from fibrous clay minerals such as sepiolite, as recently reported by Vaizoğullar [160]. The TiO_2_–ZnO@sepiolite nanoarchitecture shows good catalytic activity (ca. 85%) in the photodegradation of the antibiotic flumequine in aqueous solution. Interestingly, the sepiolite nanoarchitecture shows better degradation efficiency than the semiconducting components alone according to the following sequence [160]: TiO_2_–ZnO@sepiolite > TiO_2_–ZnO > TiO_2_ > ZnO.

It can be summarized that this type of nanostructured materials prepared from diverse clay minerals constitutes a promising way to enhance the photoactivity of the semiconductors. The idea can be extended to structurally more complex clays, as it is the case of rectorite, a 1:1 regular interstratification of two phyllosilicates (mica/montmorillonite), and the commercial clay aggregates named as LECA. Both, silicates involved in the assembly of TiO_2_–ZnO lead to photocatalysts useful for the removal of pollutants in wastewater [161–163].

Due to the abundance of clay minerals in nature, these results are relevant for the production at large scale of eco-friendly materials for depollution of water using light as energy source. Additional investigations to ascertain the observed photoactivity and to explain the involved mechanisms are still needed. Regarding possible future contributions in the use of mixed-oxide NPs, it would be also of interest to explore new synthetic procedures for the preparation of nanoparticulated TiO_2_–ZnO solids. See for instance the recent and systematic studies developed by Bachvarova-Nedelcheva et al. [164], which could be used for the assembly of diverse types of clay minerals.

**Figure 6:**
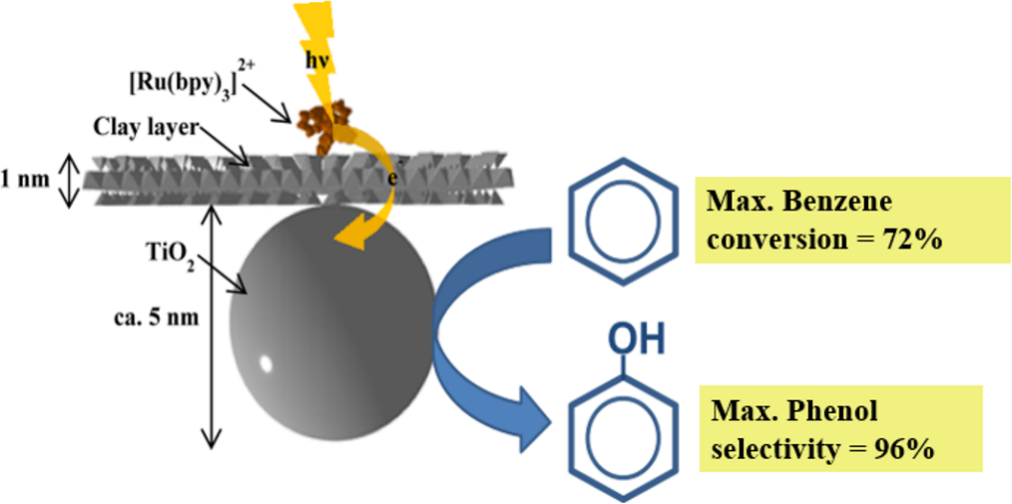
The structural arrangement of the [Ru(bpy)_3_]^2+^–TiO_2_@clay nanoarchitecture and its photocatalytic activity in the conversion of benzene to phenol. Adapted with permission from [167], copyright 2016, The Royal Society of Chemistry.

According to the Encyclopedia Britannica, the term “photosensitization” is defined as “the process of initiating a reaction through the use of a substance capable of absorbing light and transferring the energy to the desired reactants” [165]. Therefore, photosensitization represents an additional improvement of the photoactivity tuning of semiconductor–clay nanoarchitectures. For instance, tris(2,2′-bipyridine)ruthenium(II) has been used as photosensitizer for titania, being further applied to TiO_2_@clay nanoarchitectures. In this way, a synthetic saponite containing tris(2,2′-bipyridine)ruthenium(II) intercalated in the interlayer space was complexed with TiO_2_ NPs [166]. The resulting material shows enhanced stability toward visible-light irradiation, if compared with the TiO_2_ (P25) standard material photosensitized by an analogous commercially available photosensitizer (tris(2,2′ -bipyridine-4,4′-dicarboxylic acid)ruthenium(II) dichloride, abbreviated as Ru470). The stability of the two samples was compared by measuring the color change after visible-light irradiation from a solar simulator. The colour of the clay nanoarchitecture (hereafter abbreviated as [Ru(bpy)_3_]^2+^–TiO_2_@clay) did not change after irradiation for 4 h, while bleaching of Ru470 on P25 was observed. The superior stability of [Ru(bpy)_3_]^2+^–TiO_2_@clay upon the irradiation was explained as follows: [Ru(bpy)_3_]^2+^ was separated from the TiO_2_ surface by the clay nanosheet, while the photoexcited complex can still interact with TiO_2_ due to the hybrid structure (at an appropriate distance). The [Ru(bpy)_3_]^2+^–TiO_2_@clay induced the direct oxidation of aqueous benzene to phenol under visible-light irradiation (Figure 6). The oxidation of aqueous benzene to phenol was very efficient (the maximum yield of benzene conversion and the selectivity of phenol are 72 and 96%, respectively) after visible-light irradiation for 5 h. The photocatalytic reaction efficiency on the oxidation of benzene (referred to both benzene elimination yield and the selectivity of the formation of phenol) was substantially changed when the reactions were conducted in the presence of phenol (starting from a benzene/phenol mixture in water). There is a demand for an alternative to the commonly used cumene process in the production of phenol. A photocatalytic reaction with high yields and selectivity of phenol can be highly suitable for mass production [167]. The difficulties in the application to continuous-flow systems has been solved by processing the [Ru(bpy)_3_]^2+^–TiO_2_@clay nanoarchitecture as a film to be mounted in a flow reactor [168]. The film of the [Ru(bpy)_3_]^2+^TiO_2_–@clay photocatalyst is stable and can be reutilized, which is a very important advantage for the flow system. A simpler continuous-flow reactor was designed by Meshram and co-workers [169].

## Conclusions and Future Perspectives

Clay minerals are abundant, low-cost and benign materials that can be advantageous over other kinds of inorganic solids used in heterogeneous catalysis. They represent a source of components easily applicable to the development of new photocatalysts based on these silicates. We have above examined how titanium oxide and zinc oxide NPs can be assembled to diverse type of clay minerals of variable topologies leading to nanoarchitectured materials with more or less tunable photoactivity.

Optimization and streamlining of synthetic processes must be achieved in order to tailor the physical and chemical characteristics of those materials. For instance, efforts have been made to combine TiO_2_ NPs with clays and clay minerals by a simple mixing to obtain a modified catalytic activity of titanium dioxide in aqueous clay suspensions [170]. In addition, mechanochemical methods [171] seem to be applicable to the complexation of TiO_2_ with clays and clay minerals as alternative synthetic route.

Doping of TiO_2_@clay and ZnO@clay photocatalysts with different metals, particularly noble metals, as well as the use of semiconducting mixed oxides, e.g., TiO_2_/ZnO, are promising approaches, necessary to deeply study the correlations between electronic configuration, bandgap energy and photochemical efficiency. Improvements could be expected by controlled modification of the electronic characteristics, or by adding conducting polymers with different degrees of transparency, or black electronic collectors such as graphene and CNT components to the nanoarchitectured clay-based materials.

Photosensitization using organic components is a potential way to improve the photo-efficiency of these systems, which can, coupled to the beneficial effect provided by the metal doping of both semiconducting oxides, further improve the photoactivity of these new nanomaterials.

Nowadays, the main application is the removal of organic pollutants from contaminated wastewater. However, new challenges are developments regarding applications related to antibacterial activity and uses in the production of fine chemicals through photo-assisted organic syntheses. The use as films and membranes appears as a valuable alternative for industrial processes. Moreover, the clays can be used as substrates for the incorporation of additional active species, e.g, NPs or organic and biological species, for the production of multifunctional nanoplatforms as components of sensing devices and solar cells.
